# Potential usefulness of mucin immunohistochemical staining of preoperative pancreatic biopsy or juice cytology specimens in the determination of treatment strategies for intraductal papillary mucinous neoplasm

**DOI:** 10.3892/or.2013.2720

**Published:** 2013-09-05

**Authors:** TORU HISAKA, HIROYUKI HORIUCHI, SHINJI UCHIDA, HIROTO ISHIKAWA, RYUICHI KAWAHARA, YUSUKE KAWASHIMA, MASANORI AKASHI, KAZUHIRO MIKAGI, YUSUKE ISHIDA, YOSHINOBU OKABE, MASAMICHI NAKAYAMA, YOSHIKI NAITO, HIROHISA YANO, TOMOKI TAIRA, AKIHIKO KAWAHARA, MASAYOSHI KAGE, HISAFUMI KINOSHITA, KAZUO SHIROZU

**Affiliations:** 1 Department of Surgery, Kurume University School of Medicine, Kurume, Fukuoka 830-0011, Japan; 2 Department of Internal Medicine, Kurume University School of Medicine, Kurume, Fukuoka 830-0011, Japan; 3 Department of Pathology, Kurume University School of Medicine, Kurume, Fukuoka 830-0011, Japan; 4 Department of Diagnostic Pathology, Kurume University School of Medicine, Kurume, Fukuoka 830-0011, Japan; 5 Department of Surgery, Kurume University Medical Center, Kurume, Fukuoka 830-0011, Japan

**Keywords:** intraductal papillary mucinous neoplasm, morphological subtype, preoperative diagnosis, immunohistochemistry, immunocytochemistry

## Abstract

We classified resected intraductal papillary mucinous neoplasms (IPMNs) into four subtypes (gastric, intestinal, pancreatobiliary and oncocytic) according to their morphological features and mucin expression, determined their clinicopathological characteristics and investigated the possibility of preoperatively diagnosing these subtypes. Sixty resected tumors, 4 preoperative tumor biopsies and 10 preoperative pancreatic juice cytology specimens were analyzed. The gastric and intestinal types accounted for the majority of IPMNs. Non-gastric type IPMNs were of high-grade malignancy. Many of the pancreatobiliary-type IPMNs were in an advanced stage and were associated with a poor prognosis. The results of mucin immunohistochemical staining of preoperative biopsy and surgically resected specimens were in agreement with each other, and in close agreement with those for pancreatic juice cytology specimens obtained from 10 patients during endoscopic retrograde cholangiopancreatography (ERCP). The immunostaining of preoperative biopsy specimens and ERCP-obtained pancreatic juice cytology specimens may be useful in the differential diagnosis of gastric and intestinal types of IPMN. If such techniques enable the preoperative diagnosis of IPMN subtypes, their use in combination with conventional preoperative imaging modalities may lead to surgical treatment best suited for the biological characteristics of the four subtypes.

## Introduction

Intraductal papillary mucinous neoplasm (IPMN) is a cystic tumor of the pancreas, which is characterized histologically by mucin production, varying degrees of cystic dilatation of the pancreatic ducts and intrapapillary growth (1). The IPMNs account for 7.5% of clinically diagnosed pancreatic neoplasms and 16.3% of surgically resected pancreatic tumors (2). Recent advances in diagnostic imaging modalities are expected to increase the incidence of pancreatic tumors, including IPMN. In 2006, international guidelines for IPMNs were established, and a consensus was reached on an algorithm for diagnosis and treatment strategies (3). However, the methods of postoperative follow-up, bearing in mind the prognosis of IPMN patients with a long-term follow-up, choice of surgical procedures and recurrence, are largely unknown. Since the epithelial cells lining cystic lesions exhibit variable histopathological features ranging from benign to malignant, the presence of an adenomacarcinoma sequence in the carcinogenesis of pancreatic IPMN has been suggested (4). According to the international guidelines (3), patients with a suspected malignant lesion undergo surgical treatment. However, since their prognosis varies greatly depending on the histological type and degree of progression (5), preoperative diagnostic imaging and histopathological examination are of great significance in the determination of treatment strategies. Indicators suggestive of malignancy include the degree of dilation of the pancreatic duct, presence of intracystic nodules and cyst size (6). Surgical resection has been recommended if these indicators suggest the presence of a malignant lesion (3). In recent years, in addition to the histological grade of malignancy, subtype classification based on morphological characteristics has been attempted. Kimura *et al*(7) classified duodenal papilla cancer into two subtypes, and reported that there were differences in the pattern of invasion and prognosis between the two subtypes. Furukawa *et al*(8) reported a new classification of IPMNs into four subtypes (gastric, intestinal, pancreatobiliary and oncocytic types) based on their morphological characteristics and mucin expression. In addition, they reported that these subtypes differed in the location of tumor origin, histological grade of malignancy, degree of progression, prognosis and presence or absence of recurrence (9). This subtype classification, like the histological grade of malignancy, may play a large role in the determination of treatment strategies for IPMN. If information on the subtypes of IPMN is available preoperatively, its use in combination with conventional preoperative imaging modalities may lead to surgical treatment best suited for the biological characteristics of the subtypes. Therefore, we classified resected IPMNs into the four subtypes, clarified their clinicopathological characteristics, and investigated the possibility of preoperatively diagnosing these subtypes by immunohistochemical staining of preoperative biopsy or cytology specimens.

## Patients and methods

Sixty specimens that had been surgically resected in the Department of Surgery, Kurume University School of Medicine between 1996 and 2012 were examined. The resected specimens were fixed in 10% buffered formalin, and the whole specimens were cut into 5-mm slices, from which histologic sections were prepared. The sections were stained with hematoxylin and eosin (H&E), and the diameter of the tumor and presence or absence of nodules were examined histopathologically. In addition, two H&E-stained sections showing distinct morphological characteristics were selected for immunohistochemical staining.

### Criteria

**Figure 1:**
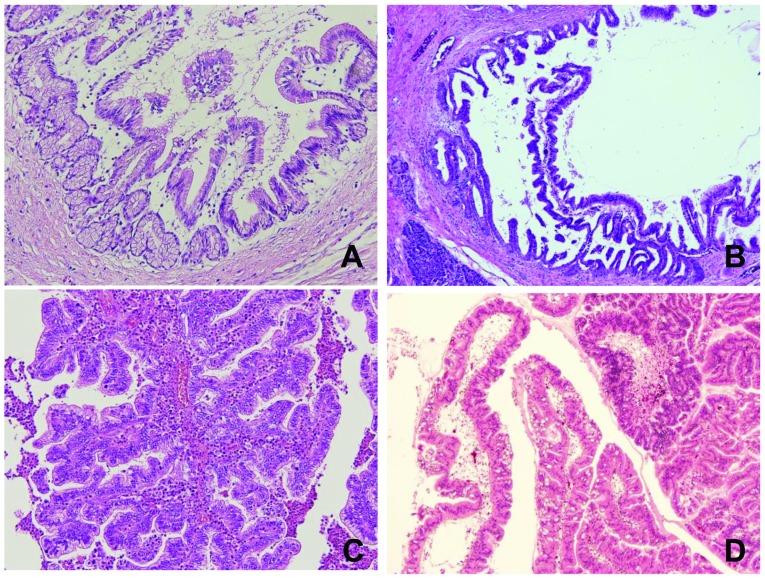
Morphological types of IPMN. (A) Gastric type; (B) intestinal type; (C) pancreatobiliary type; (D) oncocytic type.

In terms of the gross appearance, IPMNs were classified into main-duct, branch-duct and mixed types according to the already-stated criteria (5). The histological type of the tumor was determined according to the criteria of the Japan Pancreas Society (10), and classified as intraductal papillary mucinous adenoma (IPMA) or intraductal papillary mucinous carcinoma (IPMC), which was subclassified as non-invasive IPMC, minimally invasive IPMC or invasive IPMC. However, since the histopathological definition of ‘minimally invasive’ is ambiguous, all IPMNs invading beyond the epithelium were classified as invasive IPMC. Morphologically, IPMNs were classified into gastric, intestinal, pancreatobiliary and oncocytic types based on their morphology on H&E- and immunohistochemically-stained sections according to the report of Furukawa *et al*(8) (Fig. 1). At first, the subtype of the tumor was determined based on the tumor morphology on H&E-stained sections. Then, this was confirmed by immunohistochemical staining as an auxiliary method of diagnosis. When the immunohistochemical staining pattern did not correspond to the morphology observed by H&E staining, subtypes were determined based on the morphological characteristics observed by H&E staining. As has been shown to date, the gastric type is positive for MUC-5AC, but negative for MUC-1 and MUC-2; the intestinal type is positive for MUC-2 and MUC-5AC, but negative for MUC-1; the pancreatobiliary type is partially positive for MUC-1 and positive for MUC-5AC, but negative for MUC-2; and the oncocytic type is partially positive for MUC-1 and positive for MUC-5AC, but negative for MUC-2 (8,15).

### Immunohistochemical analysis

Paraffin sections were deparaffinized in xylene, rehydrated in graded alcohol and transferred to phosphate-buffered saline (PBS). Endogenous peroxidase was inactivated by incubating the sections with 0.3% hydrogen peroxide for 30 min at room temperature. Immunohistochemical staining was performed using a Vectastain ABC kit (PK-4002; Vector Laboratories, Inc., Burlingame, CA, USA). Mouse monoclonal antibody against human MUC-1 (clone Ma695, IgG1; Leica Biosystems Newcastle, Ltd., Newcastle, UK), mouse monoclonal antibody against human MUC-2 (clone Cep58, IgG1; Leica Biosystems), and mouse monoclonal antibody against human MUC-5AC (clone CLH2, IgG1; Leica Biosystems) were used as primary antibodies at a 1:100 dilution. Peroxidase Substrate kits (SK-4100; Vector Laboratories) were used for color development. The degree of immunohistochemical staining was evaluated under a light microscope at ×10 magnification. When the positive rate of tumor cells was above or below 5%, the tumor was defined as being mucin-positive and -negative, respectively.

### Attempt at diagnosis of IPMN subtypes in specimens obtained during preoperative examination

Peroral pancreatoscopy (POPS) can detect minute intraductal lesions, and is reportedly useful especially in the diagnosis of the grade of malignancy and degree of extension of IPMNs in the main pancreatic duct (11). We have been using POPS in our center since April 2011, and, in the present study, we determined IPMN subtypes in four biopsy specimens obtained by means of POPS and subjected them to H&E and immunohistochemical staining. In addition, cytology specimens obtained during preoperative endoscopic retrograde cholangiopancreatography (ERCP) were reused employing the cell transfer technique (12), and subjected to immunocytochemical staining using the same techniques as those employed for tissue sections. Since cytology specimens could not be evaluated morphologically, subtypes were determined as described for immunohistochemical evaluation.

### Statistical analysis

Fisher's exact test or the Chi-square test was used to evaluate differences between the categorical variables. The Kruskal-Wallis test was used for quantitative variables in three or more groups. Survival curves were calculated using the Kaplan-Meier method. Survival rates were compared using the log-rank test. A P-value <0.05 was considered to indicate a statistically significant result.

## Results

Surgical treatment was performed in 57 patients with IPMN, and a total of 60 lesions were examined. Of these lesions, 21, 28, 8 and 3 were of the gastric, intestinal, pancreatobiliary and oncocytic types, respectively. One patient had 2 benign lesions at the resection site: one was of the gastric type and the other was of the intestinal type. Two patients with gastric-type lesions had concomitant conventional pancreatic cancer. Two patients with intestinal-type IPMN had recurrence in the remnant pancreas 7 or 9 years after surgery, and underwent resection of the recurrent lesions, both of which were of the intestinal type. The number of patients with oncocytic-type IPMN was only 3, making inter-type comparisons difficult. However, no significant differences were observed in the gender or age among the patients with different subtypes. Of the 21 patients with gastric-type IPMN, 20 had adenoma (IPMA), but 1 had non-invasive IPMC. In contrast, >70% of patients with intestinal-type IPMN had malignant IPMN: 6 and 14 had non-invasive IPMC and invasive IPMN, respectively, and 7 had benign IPMN. The remaining 2 subtypes of IPMN exhibited malignant features: all 8 patients with pancreatobiliary-type IPMN had invasive IPMC with frequent extrapancreatic invasion. One and 2 of the 3 patients with oncocytic-type IPMN had non-invasive and invasive IPMC, respectively. There were significant differences among the percentages of histological subtypes. Among the gross classifications of IPMN, the gastric-type IPMN tended to be of the branch-duct type, and the other 3 types of IPMN were significantly more often of the main-duct type. However, some of these 3 types were classified as the branch-duct type, and, in particular, the intestinal type was classified as malignant in 3 of the 6 patients. The diameters of gastric-, intestinal-, pancreatobiliary- and oncocytic-type tumors were 14.9±6.4, 24.2±12.9, 33.2±18.6 and 23.0±9.9 mm, respectively, showing significant inter-type differences. Histological nodules were significantly more frequently observed in gastric-type than in other-type IPMNs, with no significant difference in nodule diameter. Lymph node metastases were found only in patients with invasive IPMC: in 2 of 19 patients with intestinal-type IPMN and 3 of 8 patients with pancreatobiliary-type IPMN. Two histological types of IPMC were observed at the invasive front: invasive tubular carcinoma and invasive colloid carcinoma. All pancreatobiliary and oncocytic-type IPMNs were invasive tubular carcinoma, whereas 6 and 7 of 13 IPMCs of the intestinal type were invasive tubular carcinoma and invasive colloid carcinoma, respectively. A significant difference was noted in the histological type at the invasive front between the intestinal and pancreatobiliary types (Table I).

**Figure 2:**
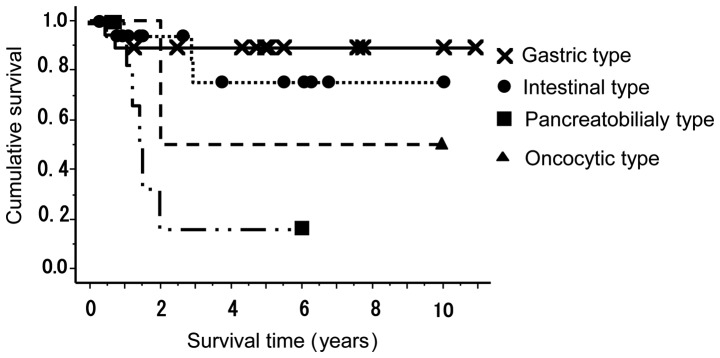
Survival curves for patients with different subtypes of IPMN. The prognosis of patients with pancreatobiliary- or oncocytic-type IPMN was significantly poorer than that of patients with gastric- or intestinal-type IPMN.

The 5-year cumulative survival rate of patients with gastric-type IPMN was 91%. However, as described above, the 2 patients with gastric-type IPMN who died had concomitant conventional pancreatic cancer, and the disease-specific survival rate was 100%. On the other hand, patients with pancreatobiliary-type IPMN had a very poor prognosis, with a 5-year cumulative survival rate of 17%, which was significantly lower than that of patients with other subtypes of IPMN. The 5-year cumulative survival rate of patients with intestinal-type IPMN was 75% (Fig. 2). No patients with adenoma or carcinoma *in situ* died of their disease.

**Figure 3:**
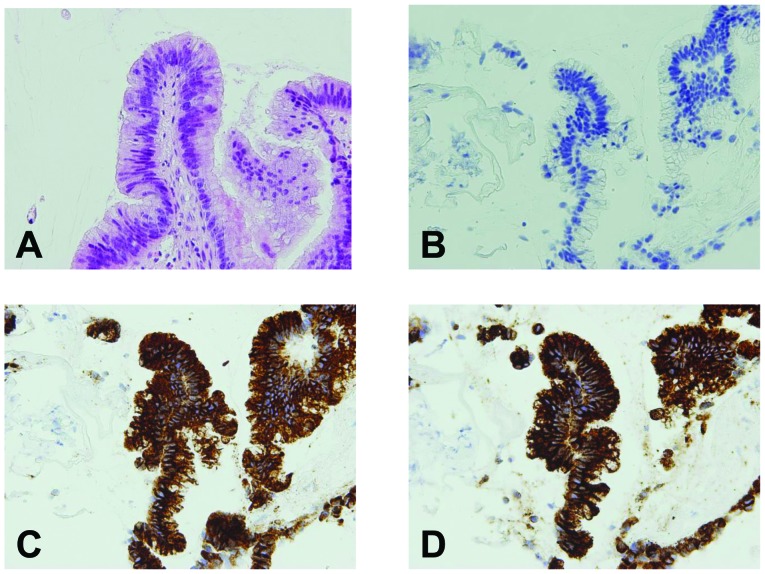
H&E and immunohistochemical staining of biopsy specimens taken during peroral pancreatoscopy (POPS). (A) H&E staining; (B) MUC-1 staining; (C) MUC-2 staining; (D) MUC-5AC staining. The lesion was negative for MUC-1 and positive for MUC-2 and MUC-5AC and was classified as the intestinal type.

**Figure 4:**
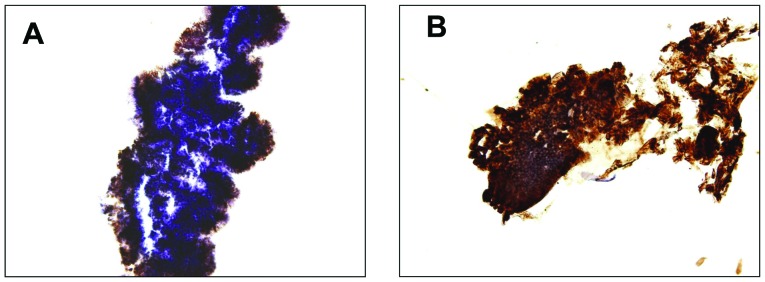
Immunocytochemical staining of pancreatic juice cytology specimens taken during endoscopic retrograde cholangiopancreatography (ERCP). (A) MUC-2 staining; (B) MUC-5AC staining. The lesion was positive for MUC-2 and MUC-5AC and was classified as the intestinal type.

Of the 4 patients who had undergone biopsies during POPS, three were diagnosed with IPMN, but one could not be diagnosed because of limited tissue availability. The biopsy specimens from the three patients were immunohistochemically positive for MUC-2 and MUC-5AC, and negative for MUC-1, suggesting intestinal-type IPMN (Fig. 3). The results of mucin immunohistochemical staining of preoperative biopsy and surgically resected specimens were in agreement with each other. In addition, cytology specimens obtained during preoperative ERCP from 10 patients were reused employing the cell transfer technique and subjected to immunocytochemical staining (Fig. 4).

The 10 patients consisted of 4 with IPMA, 2 with non-invasive IPMC and 4 with invasive IPMC. All 10 patients, except 1 with pancreatobiliary-type IPMN, had intestinal-type IPMN. Eight of these 9 patients were positive for MUC-2 and MUC-5AC. These results agreed closely with those of immunocytochemical staining. Two of them had branch duct-type IPMN. Unlike the patients positive for MUC-2 and MUC-5AC, the remaining 1 patient and the patient who was histopathologically diagnosed with pancreatobiliary-type IPMN were positive for MUC-5AC alone. On the other hand, the 4 patients histologically diagnosed with IPMA had no evidence of malignancy based on Papanicolau's classification and 3 of the 6 patients histologically diagnosed with malignant IPMN were not so diagnosed (Table II).

## Discussion

Of the 60 resected lesions, 21 and 28 were gastric- and intestinal-type IPMNs, respectively, accounting for 80% of all lesions. These results agreed closely with those previously reported (9,14), and did not greatly differ in the mean age or male-to-female ratio. These two subtypes still predominate, although recent changes in the criteria for surgical indications for IPMN have resulted in a gradual decrease in the number of surgical resections for gastric-type IPMN in our center. On the other hand, pancreatobiliary- and oncocytic-type IPMNs were often locally invasive, and a large majority of them were difficult to preoperatively differentiate from conventional pancreatic cancer. Therefore, it is debatable whether surgery should be performed for gastric- and intestinal-type IPMNs. Indeed, as described above, the percentage of malignant gastric-type IPMNs was significantly different from that of malignant intestinal-type IPMNs.

The prognosis of all patients with adenoma or carcinoma *in situ* was favorable, with a 5-year survival rate of 100% (data not shown). Therefore, it is important to completely resect a tumor in the stage of carcinoma *in situ*. Gastric-type IPMNs were mostly branch-type lesions, had smaller diameters, and less often formed nodules, which tended to be smaller in diameter, than their other-type counterparts. Most gastric-type IPMNs were benign and 91% of the resected lesions were adenomas. In addition, one lesion diagnosed as cancer was non-invasive IPMC, and no patients with gastric-type IPMN died of the primary disease, with a disease-specific survival rate of 100%. However, 2 patients with gastric-type IPMN had concomitant conventional pancreatic cancer, necessitating more careful pre- or postoperative follow-up of such patients. In the present study, one of the 2 patients was diagnosed with branch-type IPMN, and developed a conventional type of pancreatic cancer at a different site during follow-up. Yamaguchi *et al*(13) noted that 9.2% of patients with IPMN had concomitant conventional pancreatic cancer, and stated that IPMN triggered the diagnosis of early pancreatic cancer. A study reported that gastric-type IPMNs showed a significantly higher incidence of *KRAS* mutations than their intestinal-type counterparts, indicating an association with the development of conventional pancreatic cancer (14). Some conventional pancreatic cancers may develop on the basis of gastric-type IPMN. Compared with patients with gastric-type IPMN, more patients with intestinal-type IPMN had main-duct IPMN, with a higher percentage of malignant tumors. Consensus has been reached as to the need for the surgical treatment of main-duct IPMN. Indeed, many of the main-duct IPMNs are thought to be of the intestinal type. The problem here is that some branch-duct IPMNs are of the intestinal type (9,15). In the present study, 6 of the 27 intestinal-type IPMNs were of the branch-duct type, and half of them were classified as malignant. How to differentiate such IPMNs is a key point for precise treatment. Invasive cancers derived from intestinal-type IMPC were invasive tubular carcinoma or invasive colloid carcinoma at the invasive front, which were observed with the same frequency. However, histologically, no difference was noted in vascular invasion, lymph node metastasis or neural invasion (data not shown). A study by Furukawa *et al*(9) reported that intestinal-type IPMNs, many of which were invasive colloid carcinomas, were associated with a more favorable prognosis than pancreatobiliary- and oncocytic-type IPMNs, many of which were invasive tubular carcinomas. Their results are in agreement with ours. The degree of malignancy of intestinal-type IPMNs may be lower than that of IPMNs of other types except the gastric type.

All pancreatobiliary-type IPMNs were advanced carcinoma and were associated with the poorest prognosis. In this sense, this type is considered the most malignant, which is in agreement with previous reports (9,15). Only pancreatobiliary-type IPMNs were often locally invasive, and some patients with this type of IPMN underwent extended pancreatectomy or preoperative chemoradiotherapy. Since the prognosis of pancreatobiliary-type IPMN is as poor as that of conventional pancreatic cancer, it is imperative to establish an adjuvant therapy, but, to date, no effective treatment has been reported. Although its low incidence precludes a large-scale study, the treatment of this condition is an important problem that should be addressed in the future.

Although the number of oncocytic-type IPMNs was only 3, the degree of their malignancy was high and often advanced, as has previously been reported. The 2 oncocytic-type, invasive IPMNs were invasive tubular carcinoma at the invasive front. A study of a large cohort of patients with oncocytic-type IPMN reported that many of these tumors were of the oncocytic type at the invasive front (16). Although not observed in the present study, such histological features may be observed at the invasive front in a study involving a larger number of patients.

Currently, treatment strategies for IPMN are based on the presence or absence of symptoms and a comprehensive evaluation of imaging findings, as defined by the guidelines. Recently, efforts to obtain information for a decision on treatment strategies have been reported. Hirono *et al*(17) measured carcinoembryonic antigen (CEA) levels in pancreatic juice collected during preoperative ERCP, and reported that pancreatic CEA levels were high in patients with malignant tumors. Maker *et al*(18) measured mucin protein levels in intraoperatively collected pancreatic juice samples, and reported that MUC-2 and MUC-4 protein levels were elevated in patients with highly malignant tumors, indicating a close relationship between the levels of these proteins and degree of malignancy, and suggesting the usefulness of these markers for the selection of patients for surgical treatment. In the present study, the degree of malignancy depended on the subtype of the tumor, suggesting that the diagnosis of IPMN subtypes plays a major role in the determination of treatment strategies for IPMN. Therefore, we attempted to diagnose IPMN subtypes by immunohistochemical analysis of preoperative cytology specimens. Although the number of specimens was small, the diagnoses in preoperative biopsy and pancreatic juice cytology specimens were in close agreement with those in resected specimens, suggesting that these methods are very useful for the diagnosis of IPMN subtypes. These results may play a large role in the surgical treatment of IPMN. For example, surgery should be considered even for small, branch duct-type lesions on imaging, if they are of the highly malignant subtypes other than the gastric type. As also shown in the present study, the majority of highly malignant, intestinal-type IPMNs were of the main-duct type, but some of them were of the branch-duct type and malignant, making it difficult to distinguish these IPMNs by imaging alone. If these IPMNs can be distinguished by the immunocytochemical analysis of cells in the pancreatic juice collected during preoperative ERCP, it may become possible to perform more precise surgical treatment. In the present study, despite the reuse of relatively old specimens, strong MUC-2 expression was observed, which was in good agreement with the histological features. Immunocytochemically detected MUC-2-positive cells are very likely to be those of the intestinal type, as diagnosed immunohistochemically. However, the immunocytochemical stainability of MUC-1 cannot be evaluated at this point. The level of MUC-1 expression in pancreatobiliary- and oncocytic-type IPMNs, as detected by immunohistochemistry, is thought to be very low (5,15); therefore, it is expected to be fairly difficult to determine the histological type of IPMN based on the immunohistochemical expression of MUC-1. Since, in the present study, many of these two types of IPMN were malignant and advanced, we did not hesitate in the choice of surgical treatment. However, studies reported that these IPMNs were resected in the carcinoma *in situ* stage (5,15). Therefore, their diagnosis is an issue to be resolved in the future. At this point, immunocytochemistry is very useful in distinguishing gastric-type from intestinal-type IPMN. Needless to say, immunohistochemistry is difficult to perform in the case of a small pancreatic duct diameter or branch duct-type IPMNs, and immunocytochemical diagnosis may be impossible in specimens with a very small tumor component; thus, these diagnostic techniques cannot be applied to all cases. However, in recent years, examination devices in the field of pancreatic diseases have made progress, and, if thin pancreatic ducts become accessible, more precise information can be obtained.

In conclusion, we classified resected IPMNs into subtypes and clarified their clinicopathological characteristics. A high percentage of IPMNs other than the gastric type were malignant, and, in particular, many of the pancreatobiliary-type IPMNs were in an advanced stage, with a very poor prognosis. Many IPMNs classified as the main duct type on imaging were of the highly malignant subtypes other than the gastric type, and were indicated for surgery, but it should be borne in mind that some of them were of the branch-duct type and malignant. In the future, it is hoped that preoperative tests for their differential diagnosis will be established. The immunohistochemical staining of preoperative biopsy specimens and ERCP-obtained pancreatic juice may be useful in the differential diagnosis of gastric and intestinal types of IPMNs. If immunocytochemistry enables the preoperative diagnosis of IPMN subtypes, its use in combination with conventional preoperative imaging modalities may lead to surgical treatment best suited for the biological characteristics of the four subtypes.

## Figures and Tables

**Table I:** Morphological types of IPMN and clinicopathological features.

	Subtype				
		
	Gastric (21)	Intestinal (28)	Pancreatobiliary (8)	Oncocytic (3)	P-value
Gender					
Male/female	17/4	19/9	5/3	2/1	0.49
Age (years)	67.1±7.2	67.2±8.4	61.8±8.1	77.5±3.5	0.082
BD/MD/M	16/4/0	7/17/3	2/6/0	0/2/1	0.002
Tomor size (mm)	14.5±6.1	28.3±17	31.8±20	23.0±9.9	0.008
Nodule (−/+)	13/7	5/20	1/4	0/3	0.014
Nodule size (mm)	9.29±6.7	12.6±9.4	12.3±10	16.0±7.2	0.76
Histological grade					
A/NI/I	20/1/0	7/6/14	0/0/8	0/1/2	0.0001
Invasion pattern					
Tubular/colloid	0/0	6/7	8/0	2/0	0.018
Nodal stage					
pN0/pN1	21/0	26/2	5/3	3/0	0.029

BD, branch duct type; MD, main duct type; M, mixed type. A, adenoma; NI, non-invasive IPMN; I, invasive IPMN. There was no marked difference in the mean age or male-to-female ratio. Among the gross classifications of IPMNs, the gastric-type IPMN tended to be of the branch-duct type, and the other 3 types of IPMN were often of the main-duct type. Pancreatobiliary-type IPMNs had the largest diameter. Histological nodules were significantly more frequently observed in gastri-type than in other-type IPMNs, with no significant difference in nodule diameter. Lymph node metastases were found only in patients with invasive IPMC. Two histological types of IPMC were observed at the invasive front: invasive tubular and invasive colloid carcinoma.

**Table II:** Summary of the results of cytological and immunocytochemical analysis performed before surgery.

Papanicolaou's classification	MUC-1	MUC-2	MUC-5AC	Histological grade	Histologcal subtype
1. Class I	(−)	(+)	(+)	IPMA	Intestinal
2. Class I	(−)	(−)	(+)	IPMA	Intestinal
3. Class II	(−)	(+)	(+)	IPMA	Intestinal
4. Class II	(−)	(+)	(+)	IPMA	Intestinal
5. Class V	(−)	(+)	(+)	Non-invasive IPMC	Intestinal
6. Class V	(−)	(+)	(+)	Non-invasive IPMC	Intestinal
7. Class I	(−)	(+)	(+)	Invasive IPMC	Intestinal
8. Class I	(−)	(+)	(+)	Invasive IPMC	Intestinal
9. Class I	(−)	(+)	(+)	Invasive IPMC	Intestinal
10. Class V	(−)	(−)	(+)	Invasive IPMC	Pancreatobiliary

Nine of the 10 patients had intestinal-type IPMN. These results were in close agreement with those of immunohistochemical staining in that 8 (89%) of the 9 patients with intestinal-type IPMN were positive for MUC-2 and MUC-5AC.
